# Supplementary material for: Bacterial Cellulose—Carboxymethylcellulose Composite Loaded with Turmeric Extract for Antimicrobial Wound Dressing Applications
Source: Int J Mol Sci. 2023 Jan 15;24(2):1719. doi: 10.3390/ijms24021719 (PMC9864671; doi:10.3390/ijms24021719)
Supplement: Supplementary file 1 [file ijms-24-01719-s001.zip › Supplementary Material_ijms_2108242.pdf]

Supplementary Material

Bacterial Cellulose – Carboxymethylcellulose Composite Loaded with Turmeric Extract for Antimicrobial Wound Dressing Applications

1. Ultra High Performance Liquid Chromatography (UHPLC)

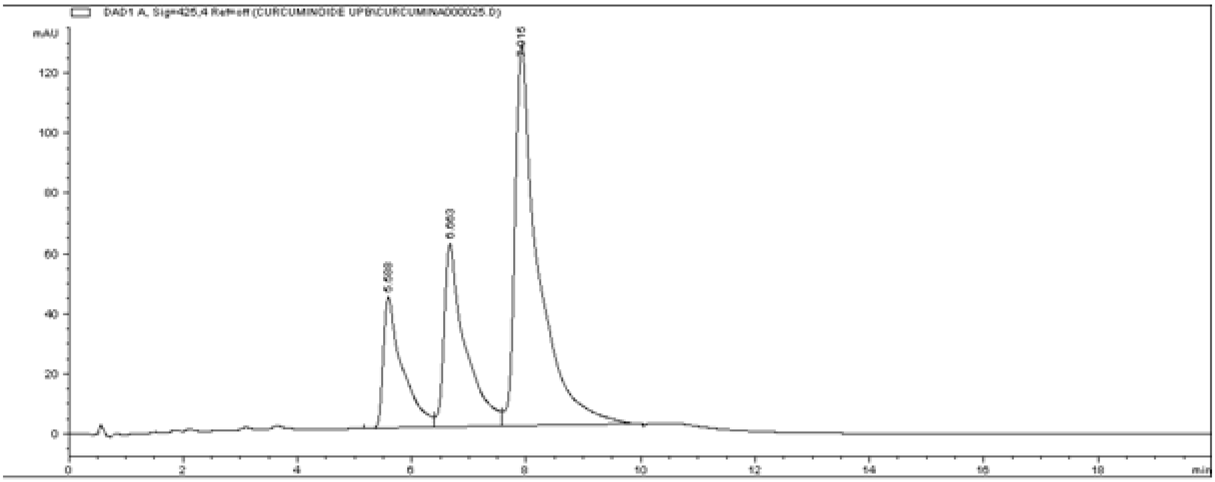

Figure S1. UHPLC chromatogram of turmeric ethanolic extracts

t_R-Retention time

Bisdemethoxycurcumin- t_R-5.588 min

Demethoxycurcumin- t_R-6.663 min

Curcumin- t_R-7.915 min

2. **Thermogravimetric analysis of the studied samples**

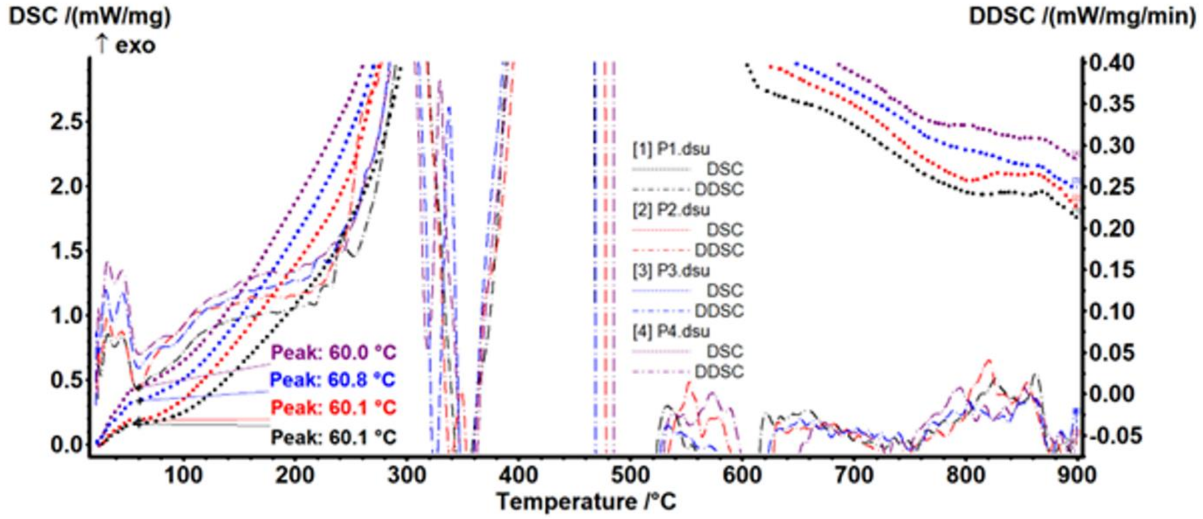

Figure S2. Detailed DSC and dDSC image of all studied samples: P1-P4.

The samples are exhibiting mass loss of 5.32-6.70% up to 200ºC and this can be attributed to the partial oxidation of organics or elimination of some residual solvent molecules. We know that an oxidation of cellulose cannot occur at such low temperature, therefore the logical choice is elimination of residual water molecules, an endothermic process.

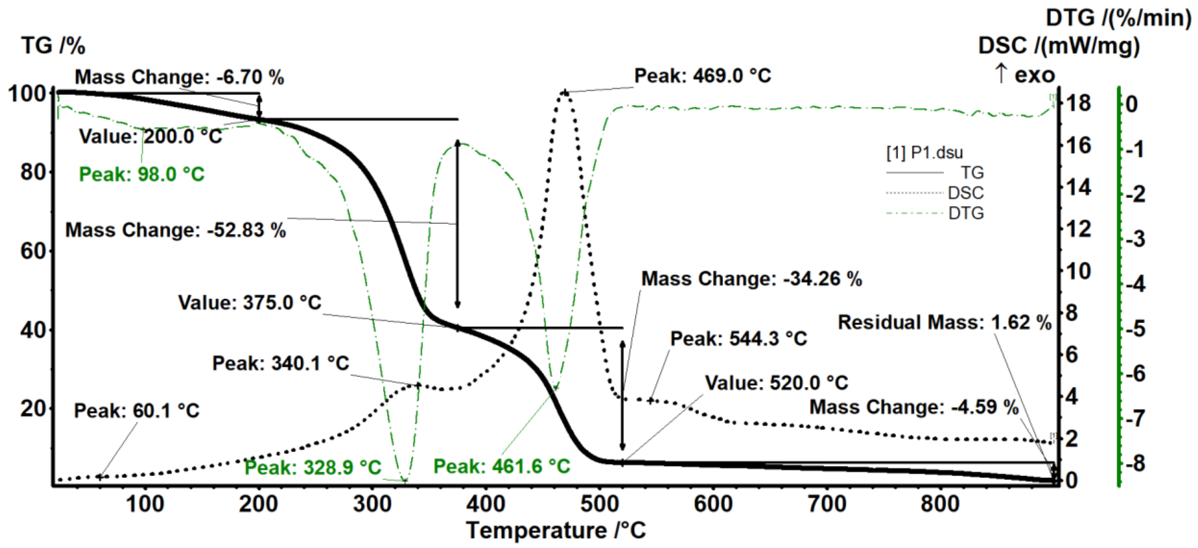

Figure S3. Thermogravimetric analysis (TG and DSC) of the sample P1.

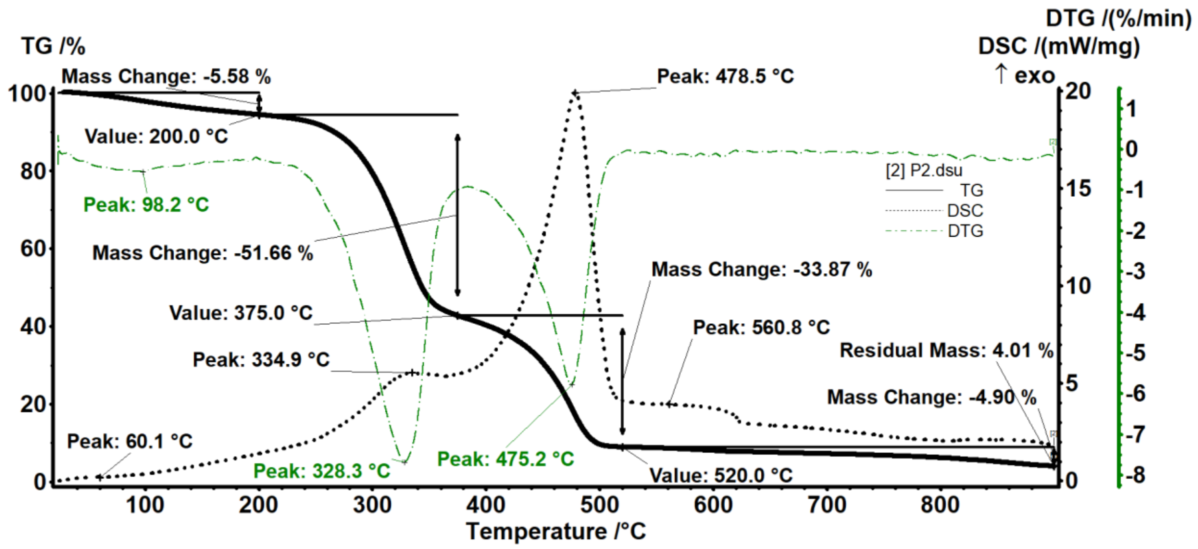

Figure S4. Thermogravimetric analysis (TG and DSC) of the sample P2.

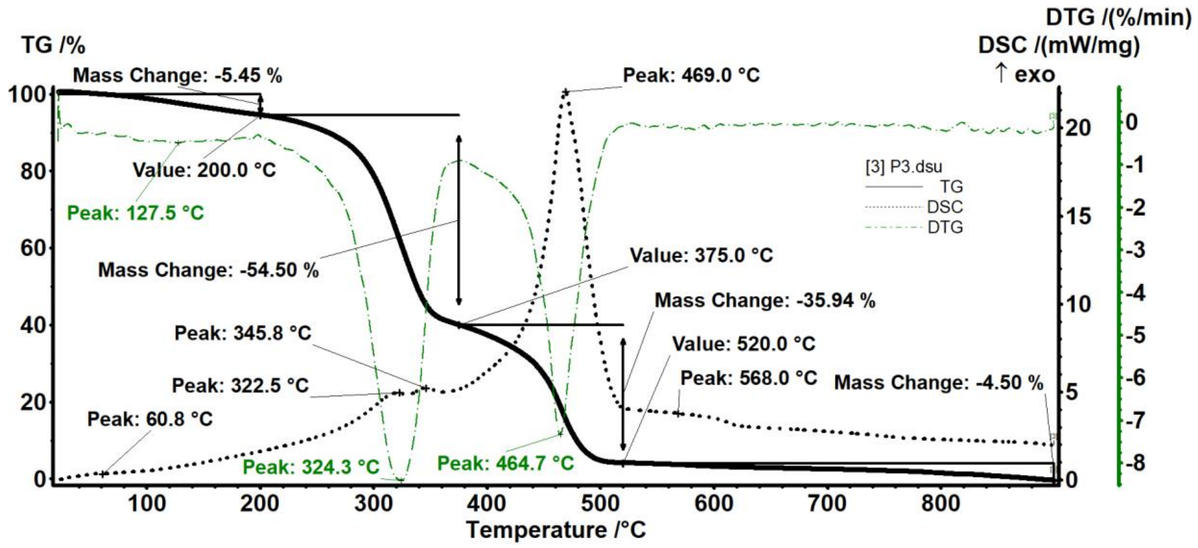

Figure S5. Thermogravimetric analysis (TG and DSC) of the sample P3.

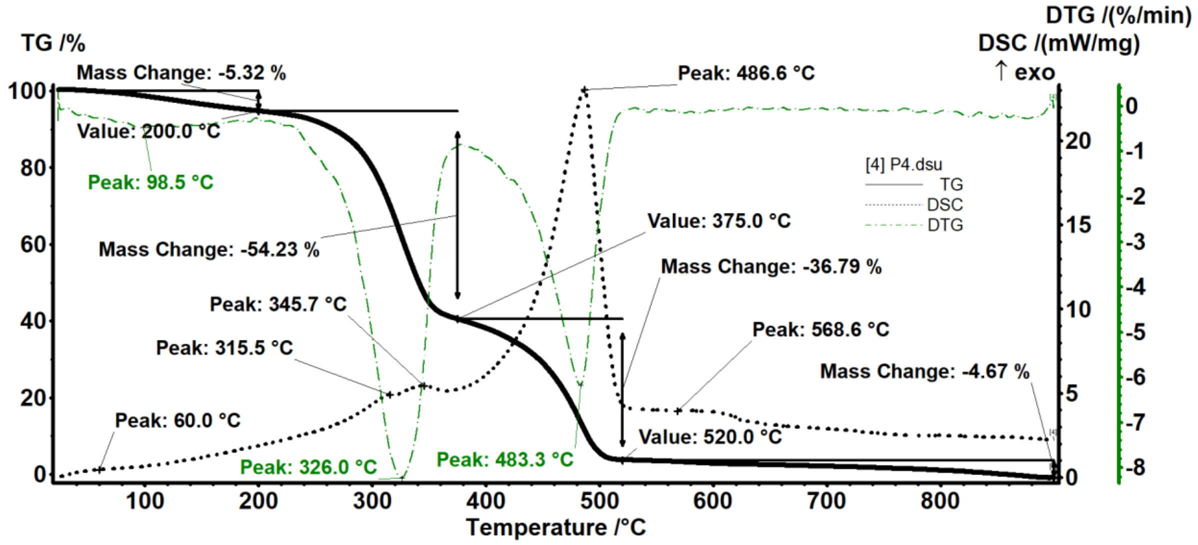

Figure S6. Thermogravimetric analysis (TG and DSC) of the sample P4.
