# Supplementary figures and images for: Bacterial Cellulose—Carboxymethylcellulose Composite Loaded with Turmeric Extract for Antimicrobial Wound Dressing Applications
Source: Int J Mol Sci. 2023 Jan 15;24(2):1719. doi: 10.3390/ijms24021719 (PMC9864671; doi:10.3390/ijms24021719)

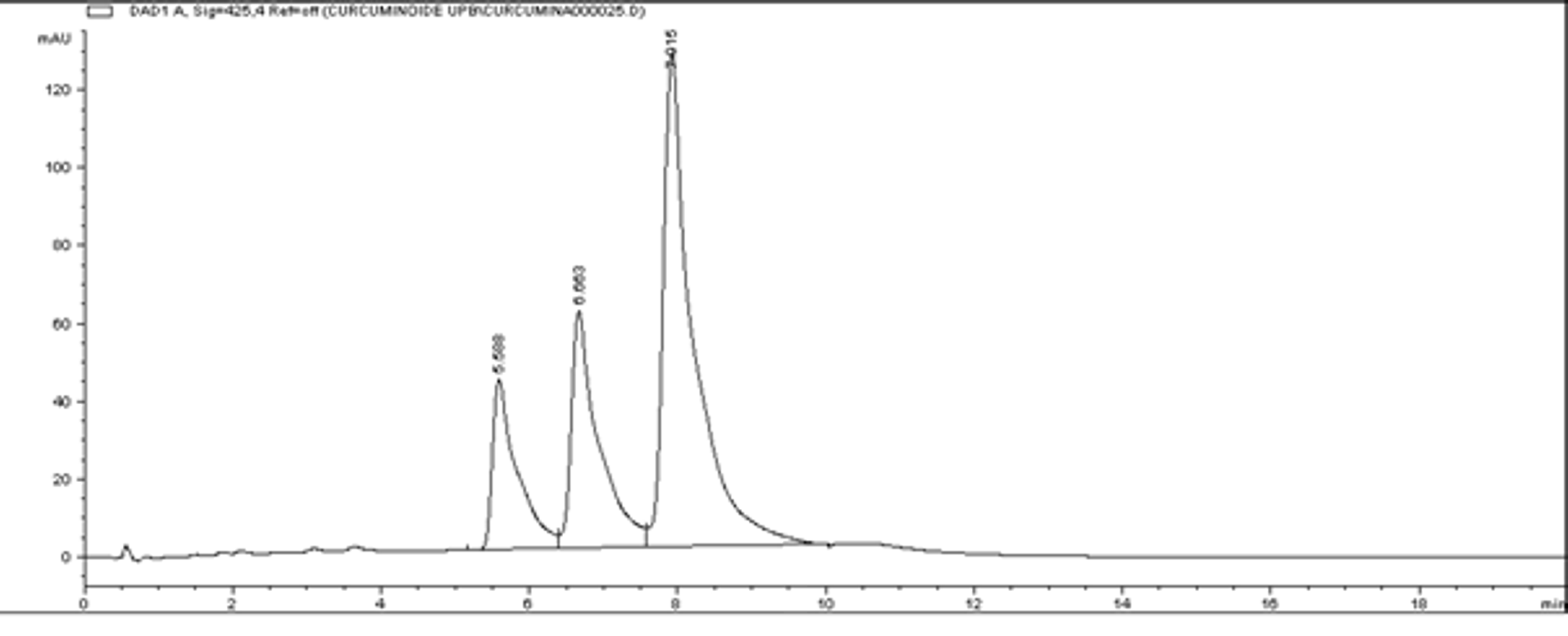

Supplement: Supplementary file 1 [file ijms-24-01719-s001.zip › Figure_S1.tif]

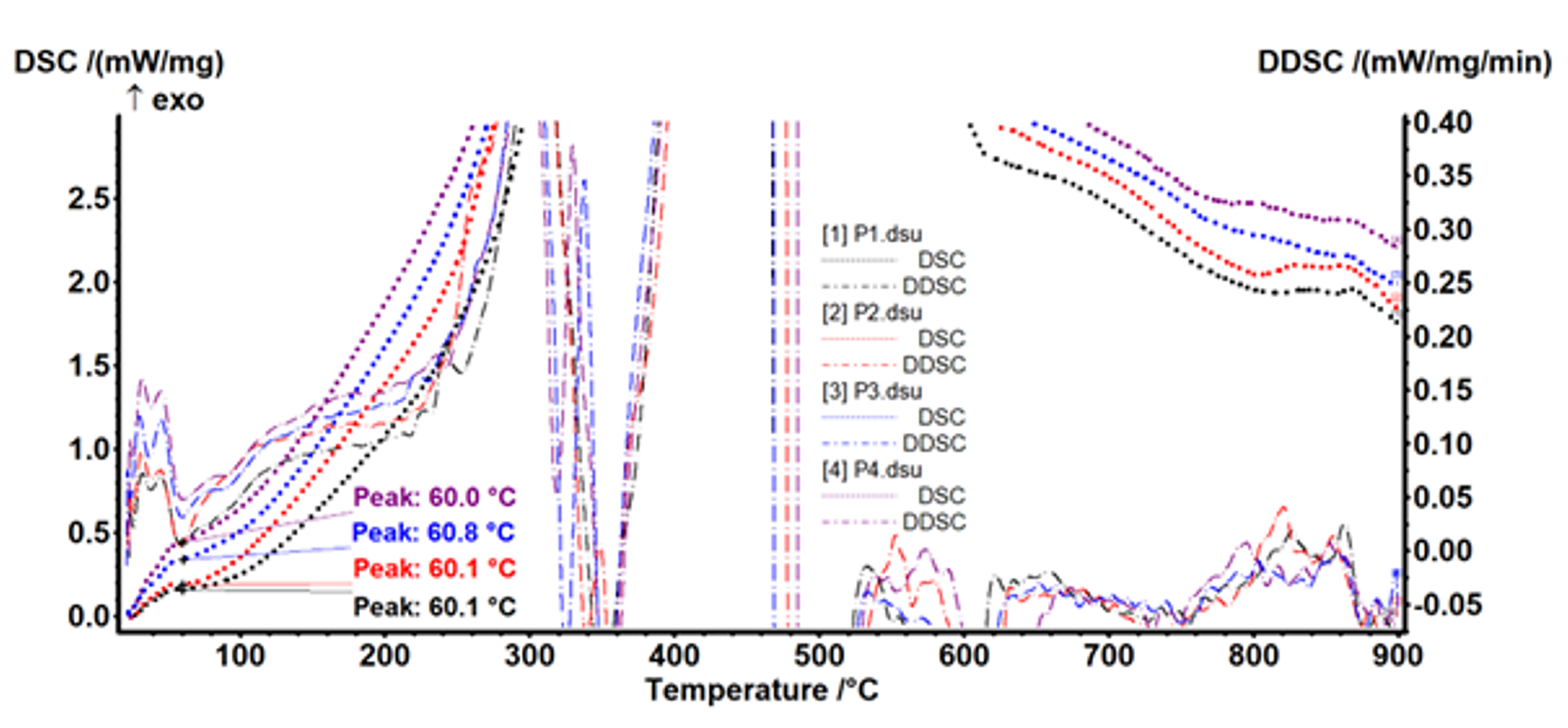

Supplement: Supplementary file 1 [file ijms-24-01719-s001.zip › Figure_S2.tif]

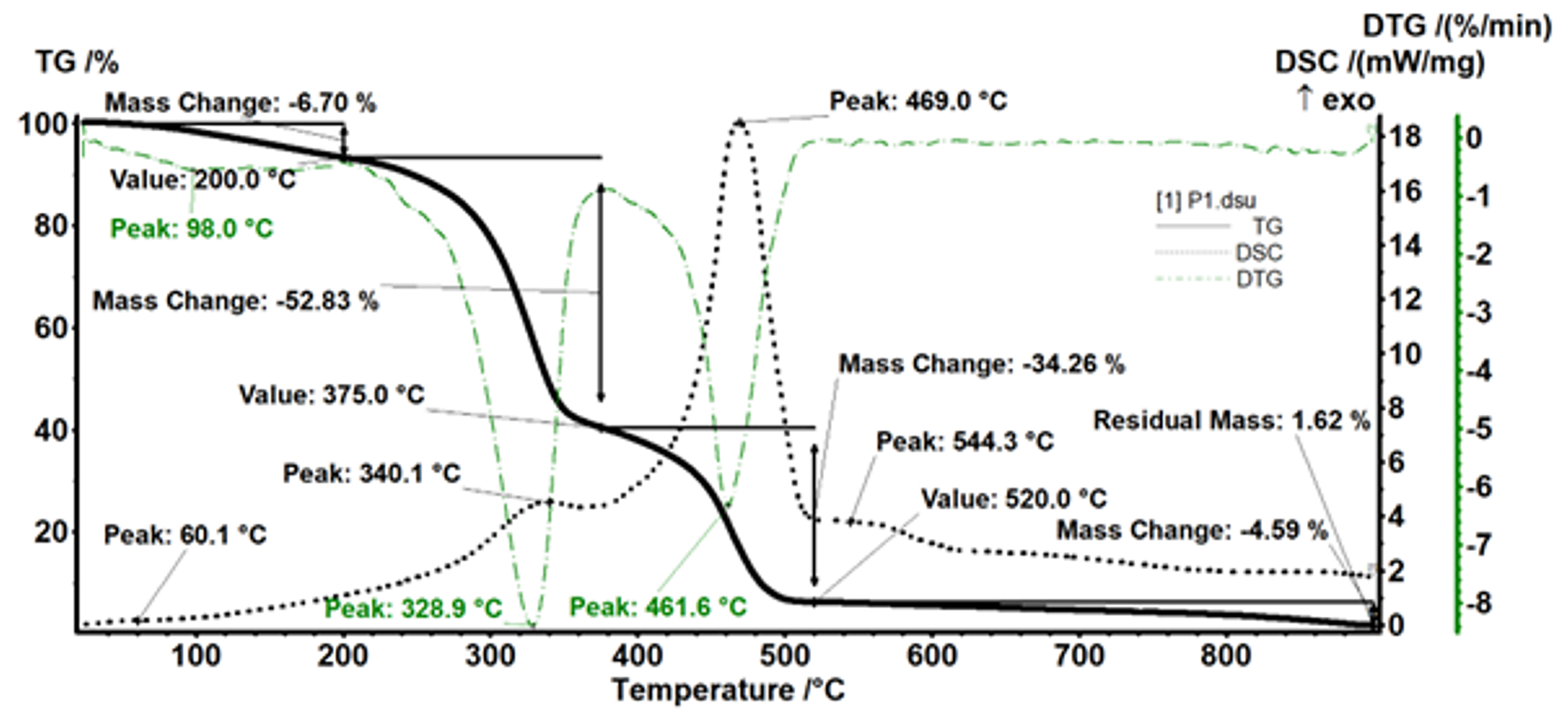

Supplement: Supplementary file 1 [file ijms-24-01719-s001.zip › Figure_S3.tif]

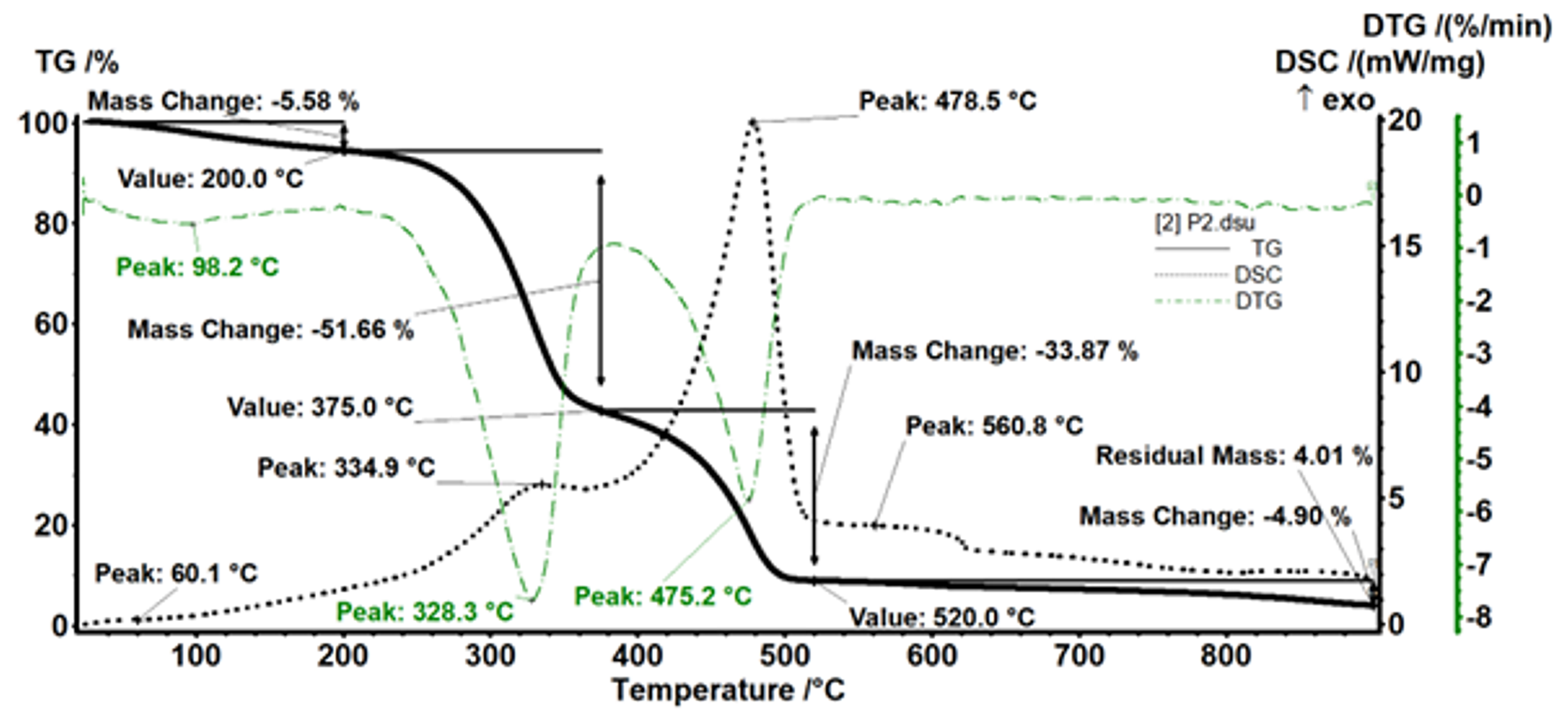

Supplement: Supplementary file 1 [file ijms-24-01719-s001.zip › Figure_S4.tif]

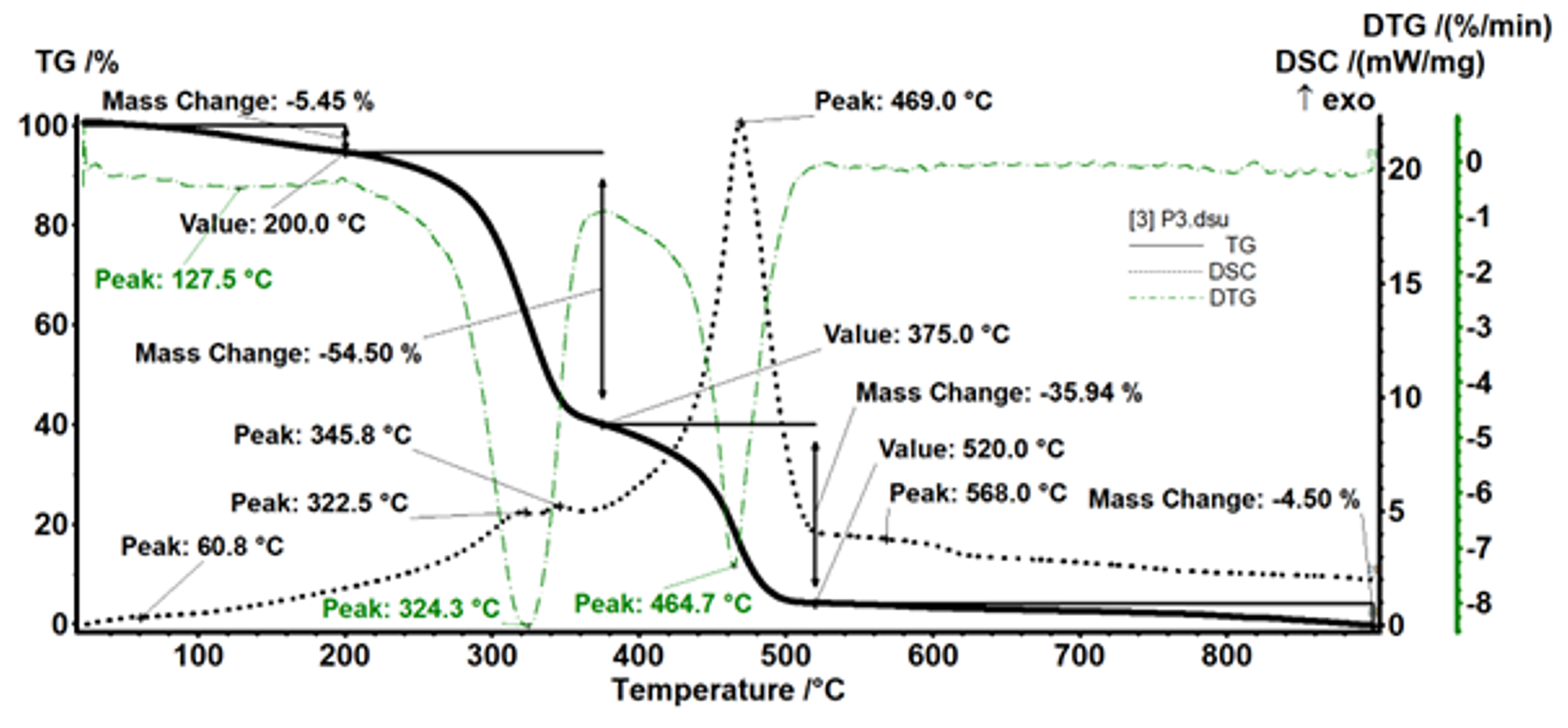

Supplement: Supplementary file 1 [file ijms-24-01719-s001.zip › Figure_S5.tif]

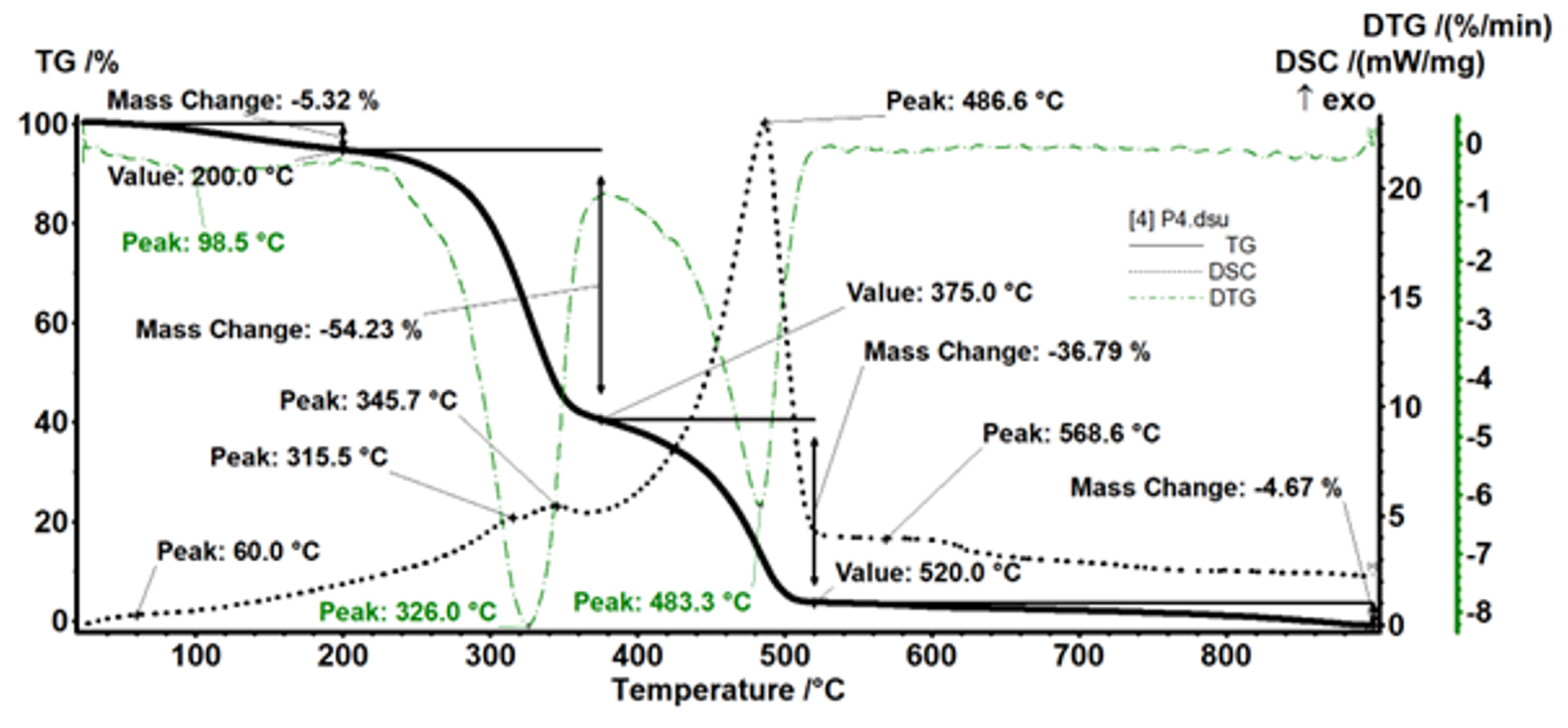

Supplement: Supplementary file 1 [file ijms-24-01719-s001.zip › Figure_S6.tif]
